# Genome-wide analysis of the R2R3-MYB transcription factor genes in Chinese cabbage (*Brassica rapa* ssp. *pekinensis*) reveals their stress and hormone responsive patterns

**DOI:** 10.1186/s12864-015-1216-y

**Published:** 2015-01-23

**Authors:** Zhen Wang, Jun Tang, Rong Hu, Peng Wu, Xi-Lin Hou, Xiao-Ming Song, Ai-Sheng Xiong

**Affiliations:** State Key Laboratory of Crop Genetics and Germplasm Enhancement, College of Horticulture, Nanjing Agricultural University, Nanjing, 210095 China; Institute of Botany, Jiangsu Province and Chinese Academy of Sciences, Nanjing, 210014 China

**Keywords:** Genome-wide analysis, R2R3-MYB transcription factor, Stress responses, Hormone signals, Chinese cabbage

## Abstract

**Background:**

The MYB superfamily is one of the most abundant transcription factor (TF) families in plants. MYB proteins include highly conserved N-terminal MYB repeats (1R, R2R3, 3R, and atypical) and various C-terminal sequences that confer extensive functions. However, the functions of most *MYB* genes are unknown, and have been little studied in Chinese cabbage.

**Results:**

Here, we analyzed 256 (55.2% of total *MYB*s) *R2R3-MYB* genes from Chinese cabbage (*Brassica rapa* ssp. *pekinensis*) and anchored them onto the 10 chromosomes and three subgenomes. The R2R3-, 3R- and atypical MYB proteins in Chinese cabbage formed 45 subgroups based on domain similarity and phylogenetic topology. Organization and syntenic analysis revealed the genomic distribution and collinear relationships of the *R2R3-BrMYB*s. Synonymous nucleotide substitution (Ka/Ks) analysis showed that the Chinese cabbage MYB DNA-binding domain is under strong purifying selection. Moreover, RNA-seq data revealed tissue-specific and distinct *R2R3-BrMYB* expression profiles, and quantitative real-time PCR (qPCR) analysis in leaves showed stress responsive expression and crosstalk with ABA-auxin signaling cascades.

**Conclusions:**

In this study, we identified the largest MYB gene family in plants to date. Our results indicate that members of this superfamily may be involved in plant development, stress responses and leaf senescence, highlighting their functional diversity.

**Electronic supplementary material:**

The online version of this article (doi:10.1186/s12864-015-1216-y) contains supplementary material, which is available to authorized users.

## Background

Plant growth and development are regulated by the coordinated expression of thousands of genes at every moment throughout their lives. Transcription factors (TFs) play a key role in these processes by self-regulating or regulating the transcription of downstream target genes. They usually consist of at least four discrete domains, namely a DNA-binding domain (DBD), a nuclear localization signal, a transcription-activation domain, and an oligomerization site [1]. These domains function together to mediate many physiological and biochemical processes, and to activate and/or repress transcription in response to endogenous and exogenous stimuli [2,3]. Additionally, most TFs are members of gene families, thereby making their regulation more complex, but also more orderly [2].

The MYB superfamily is one of the largest TF families in plants [4]. MYB proteins are found in all eukaryotes [5] and are defined by a highly conserved MYB DBD at the N-terminus [6]. The MYB domain is highly conserved among eukaryotes and forms 1–4 imperfect repeats (R0, R1, R2, and R3) with a consensus sequence of approximately 50 amino acid residues. Moreover, each repeat contains regularly spread triplet tryptophan (W) residues, forming a hydrophobic core structure [7]. The higher structure of each repeat is composed of three α-helices. The latter two helices form the HTH (helix-turn-helix) structure and bind to the promoters of target genes [6]. The third helix plays a crucial role in DNA recognition [8]. In general, these DBDs are localized to the N-terminus of MYBs, while their C-termini function as trans-acting domains (TAD) and vary considerably, which leads to the wide range of regulatory roles for the MYB gene family [9]. MYB transcription factors have been separated into four classes named 1R-, R2R3-, 3R- and 4R-MYB proteins according to the number of DBD repeats [10].

The first identified plant *MYB* gene was *C1*, isolated from *Zea mays*, and encodes a c-myb-like transcription factor that regulates anthocyanin biosynthesis [11]. An increasing number of plant R2R3-MYB superfamily members have been identified subsequently and characterized in numerous plants, such as *Arabidopsis*, grape, maize, petunia and snapdragon [4,12-14]. Plant R2R3-MYB proteins play important roles in many biological processes including cell metabolism [12,15], cell fate, development [16] and stress responses [17]. In addition, 3R-MYBs only account for a very small proportion; for example, *Arabidopsis thaliana* contains only five *3R-MYB* genes, compared with up to 190 *R2R3-MYB* and *MYB-related* genes [4].

Recently, numerous studies have shown that MYB family transcription factors play roles in plant stress responses. AtMYB15 functions as a negative regulator in the CBF pathway in response to cold stress in *Arabidopsis* [18]. *OsMYB2*, a rice MYB gene, has been shown to respond to salt, cold, and dehydration stresses [19]. The wheat *TaMYBsdu1* gene has been reported to act as a potentially important regulator in tolerance to salt and drought stresses [20]. *AmMYB1* from *Avicennia marina* regulates the response processes under salt stress and transgenic tobacco plants expressing it showed better tolerance to NaCl stress [21]. Wang *et al*. have reported that transferring apple *MdSIMYB1* to both tobacco and apple could increase tolerance to multiple stresses [22].

R2R3-MYB family transcription factors participate in multiple plant-specific processes, raising the hypothesis that their expansion may be responsible for the diversity of plant evolution [2]. R2R3-MYB families from several sequenced plants such as *Arabidopsis*, rice, corn, wheat, barley and soybean have been identified [4,13,23,24]. However, studies on R2R3-MYB TFs from vegetable crops have been limited and unsystematic so far. Chinese cabbage (*Brassica rapa* ssp. *pekinensis*) is a vital Cruciferae *Brassica* vegetable, but the functions of only a few Chinese cabbage R2R3-MYB (R2R3-BrMYB) genes (MYBs) are known [25]. Therefore, it is very important to characterize the roles of R2R3-BrMYBs and to achieve complete identification and classification of these genes. In this study, we first identified 256 MYB family members in Chinese cabbage and then systematically analyzed their organization, collinearity and stress-responsive expression patterns. Our results showed the functional diversity of the R2R3-BrMYB genes, which may be involved in plant development, stress responses and leaf senescence.

## Results and discussion

### Identification and conserved DBD analysis of MYB TFs in Chinese cabbage

**Figure 1:**
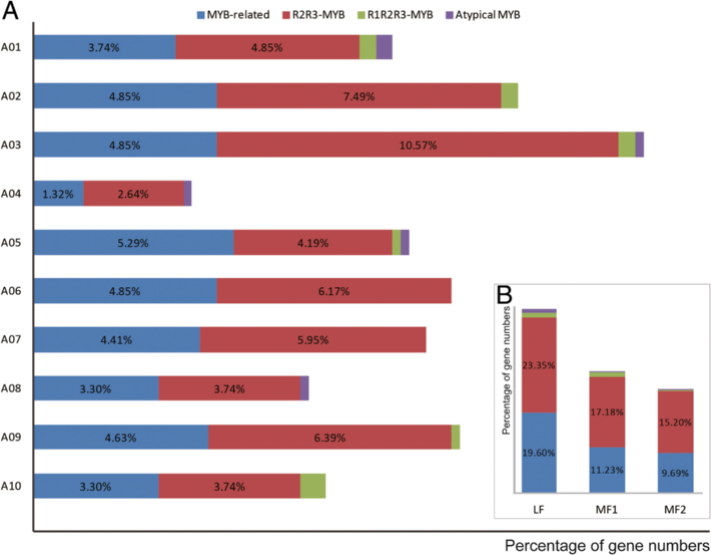
**Chromosomal distribution of MYB transcription factor genes.** The proportion of each class distributed among 10 chromosomes **(A)** and three subgenomes **(B)**. We classified BrMYB transcription factors into four distinct groups, namely MYB-related, R2R3-MYB, R1R2R3-MYB, and atypical MYB, based on the presence of one, two, three and more than three MYB repeats, respectively.

To define the BrMYB gene family, we searched the entire *B. rapa* genome sequence for genes containing the MYB domain using the Pfam program with the MYB DBD model (PF00249) as a query. We identified more than 400 sequences containing MYB or MYB-like repeats (Additional file 1: Table S1). Firstly, 21 Golgi-associated retrograde protein (GAPRs) were excluded [26]; consequently, based on the identification numbers and chromosome locations, any redundant sequences were removed from the dataset. To verify the reliability of our results, we also performed SMART analysis to identify all of the putative MYB protein sequences in the Chinese cabbage genome. The results were consistent with the Pfam outcome. Finally, 191 MYB-related, 256 typical R2R3-MYB (2R-MYB) (including 3 *AtCDC5* homologous genes) and 11 R1R2R3-MYB (3R-MYB) proteins were successfully identified in Chinese cabbage. Six atypical MYB proteins were also identified, including four 4R-like proteins and two 5R-MYB proteins [24]. The resulting sequences were named according to the standard constructed by Stracke [27], and the corresponding relationships between the names we defined and their genomic IDs are shown in Additional file 1: Table S1. Our analysis revealed that the R2R3-MYB subfamily was the largest MYB subgroup, comprising 55.73% of Chinese cabbage MYB genes (Figure 1), which was consistent with previous studies in rice and *Arabidopsis* [4,24].

**Figure 2:**
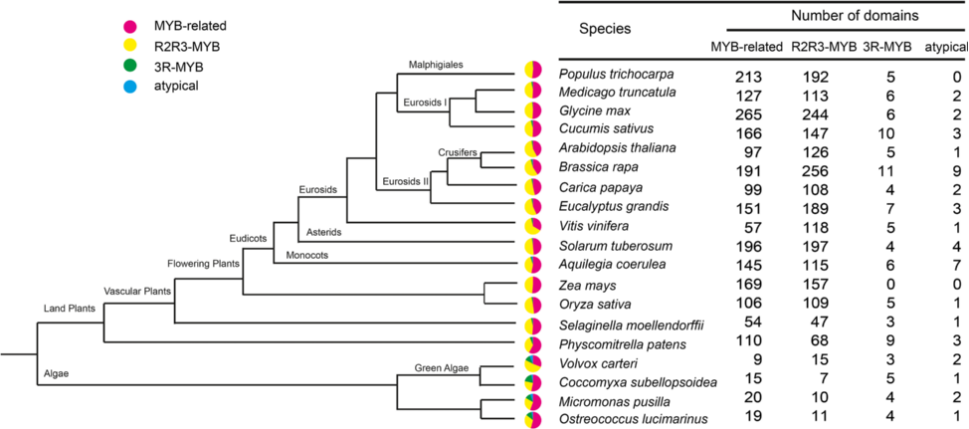
**MYB transcription factor comparisons among different species.** Different colors represent each family domain in the MYB superfamily. The colored sections represent the number of transcription factor domains identified in each species. Gray represents the absence of a domain.

Wang *et al*. divided the Chinese cabbage genome into three subgenomes according to their fractionation degree, namely the least fractionated (LF), medium fractionated (MF1), and most fractionated (MF2) subgenomes, and the LF subgenome seemed to be fractionated later than the MF1 and MF2 subgenomes because that the earlier subgenomes evolutionally appeared, the more time they would have to proceed fractionation [28]. In our study, the LF subgenome had the highest number of MYB genes (43.97%), and atypical *MYB* genes were distributed in all three subgenomes (Figure 1B), indicating that atypical *MYB* genes appeared before the MF subgenomes began to fractionate. In total, *MYB* genes represented approximately 1.1% of the 41,174 predicted Chinese cabbage protein-coding loci. We also counted *MYB* genes in plants ranging from algae to higher plants, except *P. trichocarpa* [23], *G. max* [29], *A. thaliana* [4], *V. vinifera* [14], *Z. mays* [13] and *O. sativa* [30] that had published MYB information, while there were few genome-wide studies of MYBs in other selected plants, thus MYB numbers in these plants were obtained through the strategy used in Chinese cabbage MYB identification in this study (Figure 2); among these species, land plants seemed to carry far more *MYB* genes than algae, indicating that a huge expansion of MYB family members occurred after the evolution of land plants. The R2R3-MYB family is the most abundant transcription factor family in most plants, with 130 members in *Arabidopsis* [27], 141 in rice [24,31], and 118 in grape [14]. Moreover, species-specific members of this subgroup of the MYB gene superfamily have been identified.

**Figure 3:**
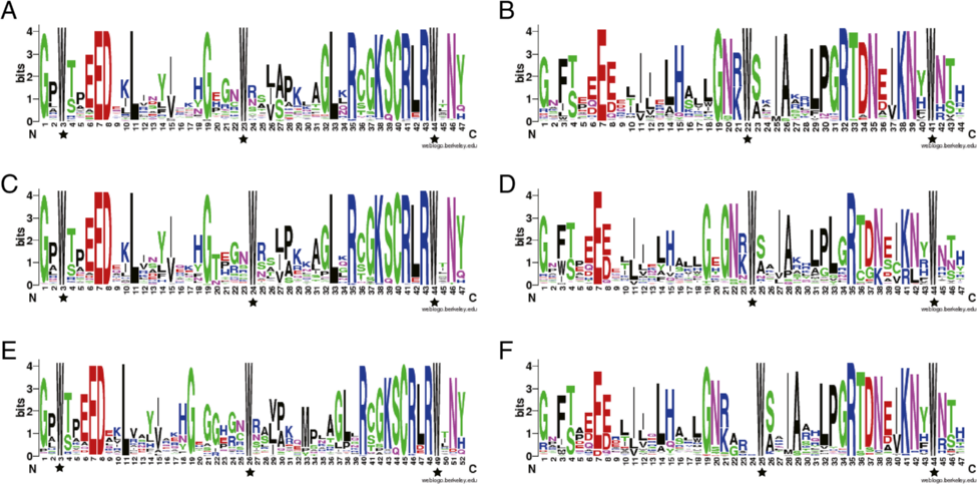
**Comparison of DNA-binding domains of R2R3-MYB transcription factor proteins in** ***Arabidopsis*** **, Chinese cabbage and rice. (A)**, **(C)**, and **(E)** represent the R2 repeats, and **(B)**, **(D)**, and **(F)** represent the R3 repeats of R2R3-MYBs in *Arabidopsis*, Chinese cabbage and rice, respectively. Highly conserved tryptophan amino acids are labeled with asterisks.

To investigate the homologous domain sequence features, we performed multiple alignment analysis using the 130, 256 and 141 homologous domain amino acid sequences of R2R3 repeats from *Arabidopsis*, Chinese cabbage and rice, respectively (Figure 3). The basic regions of the MYB domains had around 103 amino acid residues, with rare deletions or insertions as previously reported [32]. Figure 3 shows the distribution of amino acid residues at the corresponding positions of the R2 and R3 MYB repeats of each species. Generally speaking, the distribution of conserved amino acids among the MYB domains of Chinese cabbage was very similar to those of *Arabidopsis* and rice, suggesting evolutionary conservation of MYBs among plants. They all included highly conserved triplet tryptophan (Trp, W) residues in each DBD repeat, and the characteristic W residues were located at positions 3, 23, and 44 of the R2 repeat (Figure 3C) and 3, 24 and 44 of the R3 repeat in Chinese cabbage (Figure 3C,D); similar localization was observed in both *Arabidopsis* and rice (Figure 3A,B,E and F). Conserved W residues have also been found in *MYB-related* and *3R-MYB* genes (Additional file 2: Figure S1), indicating the indispensable role of these residues in maintaining the helix-turn-helix structure of MYB domains [33]. In the R3 repeat, the first tryptophan (Trp3) residue was generally replaced by phenylalanine (Phe, F). However, the second and the third tryptophan residues were apparent and showed high conservation. In each repeat, the major conserved residues in the MYB domain were mainly distributed at the second and third conserved Trp residues, suggesting that the first part of each repeat in the MYB domain was apparently less conserved [34]. This was mainly because helix-3 is highly conserved in Chinese cabbage for its DNA recognition and direct contact functions. In addition to the highly conserved W residues, more than 90% of alternative residues were highly conserved in the Chinese cabbage R2R3-MYB domains, including E-7, D-8, L-11 and G-19 in R2 repeats and G-1, E-7, G-19, G-21, N-22 and R-35 in R3 repeats (Figure 3). However, the MYB domains in both repeats of the *R2R3-MYB* genes in Chinese cabbage and rice seemed to be larger than that in Arabidopsis ones; this was inferred from the space between neighboring W residues. An analogous phenomenon also existed in other types of MYB domains (Additional file 2: Figure S1). The largest insertions in MYB domains were observed in rice, while the size varied only slightly between *Arabidopsis* and Chinese cabbage.

### Chromosomal distribution and collinearity analysis of duplicated *R2R3-BrMYB* genes

**Figure 4:**
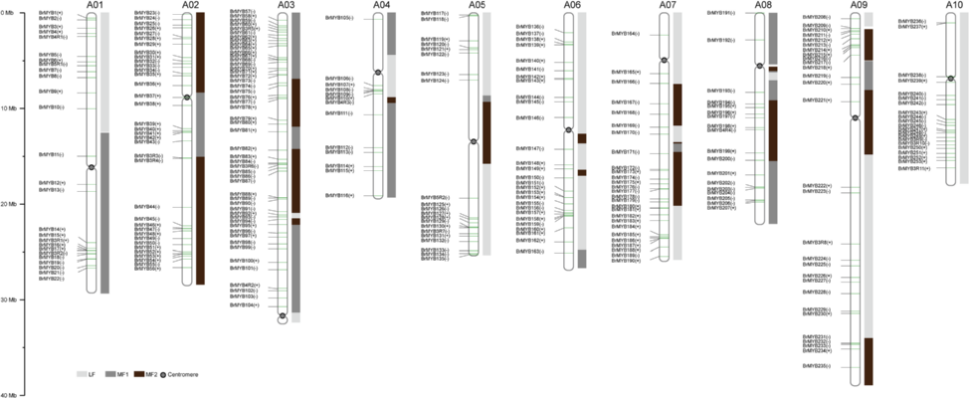
**Distribution of** ***R2R3-MYB*** **genes on the 10 chromosomes and three subgenomes of Chinese cabbage.** The different colored blocks represent different subgenomes. *R2R3-BrMYB* genes are shown on the left of each chromosome. The positive (+) and negative (−) symbols following each gene represent forward and reverse orientations, respectively, on the chromosome or subgenome. Gene positions and the size of each chromosome can be estimated using the scale on the left of the figure.

Genome chromosomal location analysis revealed that the Chinese cabbage *MYB*s were distributed on all 10 chromosomes and all three subgenomes (Figure 4 and Additional file 3: Figure S2). In total, 273 *BrMYB*s (256 MYB-type ones and 17 members contain MYB domains > 2) were separately mapped onto chromosomes A01–A10, except for three members (*BrMYB254*, *BrMYB255* and *BrMYB256*) on the scaffolds. On average, one *R2R3-MYB* gene was present every 2.5 Mb relative to the whole genome. Relatively high densities of *BrMYB*s were observed in some chromosomal regions, including the top and bottom of chromosomes A01, A02, A03, A05, A06 and A09, and the bottom of chromosomes A04, A07, A08 and A10. In contrast, almost all central chromosome regions lacked *R2R3-MYBs*. Among the 10 chromosomes, chromosome A03 contained the most *R2R3-MYB* genes, while chromosome A04 possessed the least (~5%) (Figure 1A). Furthermore, the 273 *BrMYB* genes were also mapped onto the chromosomes in relation to the three subgenomes (LF, MF1, and MF2), including 116 in LF, 85 in MF1, and 72 in MF2 (Figure 1B and Figure 3). Therefore, the 273 putative R2R3 proteins (including R3- and atypical MYBs), could be divided into three groups accordingly. However, *3R-MYB* and atypical *MYB* genes were seemingly not present on all chromosomes in Chinese cabbage; furthermore, chromosomes A06 and A07 only had *MYB-related* and *R2R3-MYB* genes (Figure 4 and Additional file 3: Figure S2).

**Figure 5:**
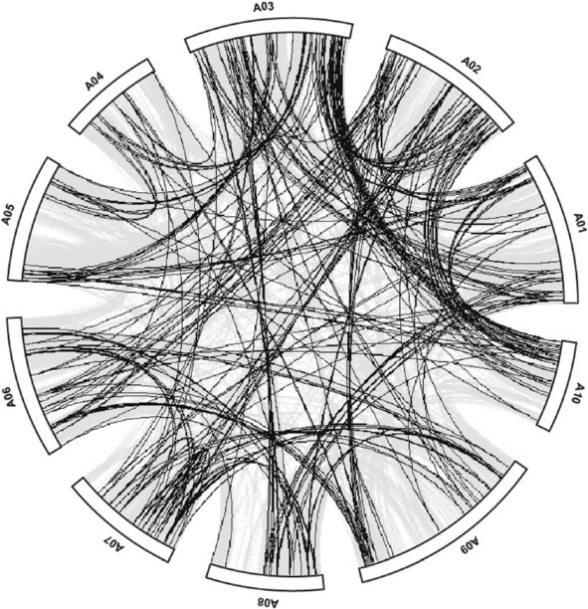
**Depiction of duplicated R2R3-BrMYB genes on the 10 Chinese cabbage chromosomes.** Grey lines indicate collinear blocks in the whole Chinese cabbage genome, and black lines indicate duplicated *R2R3-MYB* gene pairs.

It has been confirmed that gene duplication occurred during the process of plant evolution, thereby contributing to the establishment of new gene functions [35]. The emergence of multigene families is attributed to gene duplication via region-specific duplication or genome-wide polyploidization. MCScanX was used to further analyze the collinear relationships of the *R2R3-MYBs* in Chinese cabbage [36]. It had been well addressed that a genome duplication event in Chinese cabbage occurred approximately 5–9 million years ago (MYA) and resulted in a highly duplicated genome [28]. The collinear relationships of the duplicated pairs in the R2R3-MYB gene family in Chinese cabbage are shown in Figure 5. In total, we identified 185 pairs (pairs and groups of three or more) of highly similar paralogs that shared a high degree of identity through their protein sequences (Table 1 and Figure 5). At least eight *BrMYB*s were located in duplicated segments on each chromosome. Interestingly, all of the *R2R3-MYB* genes in subgenome LF had one or more duplicates in the other subgenomes, suggesting that all *R2R3-MYB* genes were retained in the genomic triplication of Chinese cabbage; this might also have contributed to the expansion of *R2R3-MYB* gene family.

**Table 1:** **The selection and divergence of R2R3 type MYB duplication genes in Chinese cabbage**

**Duplication pair**	**Duplicated gene 1**			**Duplicated gene 2**			**Ka**	**Ks**	**Ka/Ks**	**Purification selection**	**Divergence time (MYA)**
	**Gene name**	**Chromosome**	**Subgenome**	**Gene name**	**Chromosome**	**Subgenome**					
BrMYB1-BrMYB56	BrMYB1	A01	LF	BrMYB56	A02	MF1	0.45	2.35	0.19	YES	7.83
BrMYB3-BrMYB91	BrMYB3	A01	LF	BrMYB91	A03	MF2	0.2	0.93	0.22	YES	3.11
BrMYB8-BrMYB99	BrMYB8	A01	LF	BrMYB99	A03	MF1	0.09	0.28	0.33	YES	0.93
BrMYB9-BrMYB100	BrMYB9	A01	LF	BrMYB100	A03	MF1	0.17	0.45	0.38	YES	1.49
BrMYB4R1-BrMYB4R2	BrMYB4R1	A01	LF	BrMYB4R2	A03	MF1	0.1	0.25	0.41	YES	0.84
BrMYB4-BrMYB102	BrMYB4	A01	LF	BrMYB102	A03	MF1	0.24	0.37	0.65	YES	1.23
BrMYB3-BrMYB103	BrMYB3	A01	LF	BrMYB103	A03	MF1	0.09	0.55	0.17	YES	1.83
BrMYB9-BrMYB73	BrMYB9	A01	LF	BrMYB73	A03	MF1	0.25	0.79	0.32	YES	2.64
BrMYB7-BrMYB98	BrMYB7	A01	LF	BrMYB98	A03	MF1	0.08	0.48	0.18	YES	1.59
BrMYB21-BrMYB133	BrMYB21	A01	MF1	BrMYB133	A05	LF	0.06	0.23	0.27	YES	0.76
BrMYB22-BrMYB134	BrMYB22	A01	MF1	BrMYB134	A05	LF	0.08	0.21	0.38	YES	0.68
BrMYB14-BrMYB125	BrMYB14	A01	MF1	BrMYB125	A05	LF	0.08	0.24	0.33	YES	0.81
BrMYB1-BrMYB149	BrMYB1	A01	LF	BrMYB149	A06	LF	0.52	2.61	0.2	YES	8.72
BrMYB3-BrMYB163	BrMYB3	A01	LF	BrMYB163	A06	MF1	0.34	3.85	0.09	YES	12.83
BrMYB4R1-BrMYB4R4	BrMYB4R1	A01	LF	BrMYB4R4	A08	MF2	0.1	0.27	0.36	YES	0.89
BrMYB4-BrMYB198	BrMYB4	A01	LF	BrMYB198	A08	MF2	0.06	0.39	0.16	YES	1.29
BrMYB7-BrMYB196	BrMYB7	A01	LF	BrMYB196	A08	MF2	0.09	0.42	0.21	YES	1.39
BrMYB8-BrMYB197	BrMYB8	A01	LF	BrMYB197	A08	MF2	0.09	0.31	0.27	YES	1.04
BrMYB7-BrMYB222	BrMYB7	A01	LF	BrMYB222	A09	LF	0.2	1.47	0.13	YES	4.91
BrMYB24-BrMYB16	BrMYB24	A02	MF2	BrMYB16	A01	MF1	0.62	2.23	0.28	YES	7.43
BrMYB36-BrMYB72	BrMYB36	A02	MF2	BrMYB72	A03	MF1	0.08	0.34	0.23	YES	1.15
BrMYB48-BrMYB155	BrMYB48	A02	MF1	BrMYB155	A06	LF	0.11	0.2	0.54	YES	0.67
BrMYB40-BrMYB142	BrMYB40	A02	MF1	BrMYB142	A06	LF	0.32	1.09	0.3	YES	3.63
BrMYB39-BrMYB184	BrMYB39	A02	MF1	BrMYB184	A07	LF	0.05	0.28	0.19	YES	0.93
BrMYB40-BrMYB185	BrMYB40	A02	MF1	BrMYB185	A07	LF	0.1	0.29	0.35	YES	0.97
BrMYB43-BrMYB189	BrMYB43	A02	MF1	BrMYB189	A07	LF	0.07	0.33	0.21	YES	1.1
BrMYB39-BrMYB204	BrMYB39	A02	MF1	BrMYB204	A08	MF1	0.23	1.93	0.12	YES	6.45
BrMYB40-BrMYB203	BrMYB40	A02	MF1	BrMYB203	A08	MF1	0.27	1.01	0.27	YES	3.38
BrMYB41-BrMYB202	BrMYB41	A02	MF1	BrMYB202	A08	MF1	0.38	1.92	0.2	YES	6.42
BrMYB56-BrMYB218	BrMYB56	A02	MF1	BrMYB218	A09	MF2	0.08	0.29	0.27	YES	0.95
BrMYB48-BrMYB212	BrMYB48	A02	MF1	BrMYB212	A09	MF2	0.12	0.3	0.41	YES	0.99
BrMYB39-BrMYB233	BrMYB39	A02	MF1	BrMYB233	A09	MF2	0.25	3.41	0.07	YES	11.36
BrMYB85-BrMYB18	BrMYB85	A03	MF2	BrMYB18	A01	MF1	0.09	0.31	0.3	YES	1.04
BrMYB97-BrMYB6	BrMYB97	A03	MF1	BrMYB6	A01	LF	0.07	0.36	0.2	YES	1.21
BrMYB94-BrMYB11	BrMYB94	A03	MF2	BrMYB11	A01	MF1	0.11	0.44	0.25	YES	1.45
BrMYB88-BrMYB13	BrMYB88	A03	MF2	BrMYB13	A01	MF1	0.09	0.38	0.24	YES	1.26
BrMYB90-BrMYB12	BrMYB90	A03	MF2	BrMYB12	A01	MF1	0.09	0.25	0.36	YES	0.84
BrMYB63-BrMYB22	BrMYB63	A03	MF1	BrMYB22	A01	MF1	0.2	1.3	0.16	YES	4.33
BrMYB64-BrMYB21	BrMYB64	A03	MF1	BrMYB21	A01	MF1	0.15	0.78	0.2	YES	2.59
BrMYB96-BrMYB5	BrMYB96	A03	MF1	BrMYB5	A01	LF	0.09	0.31	0.3	YES	1.04
BrMYB3R6-BrMYB3R2	BrMYB3R6	A03	MF2	BrMYB3R2	A01	MF1	0.18	0.36	0.5	YES	1.19
BrMYB57-BrMYB3R1	BrMYB57	A03	MF1	BrMYB3R1	A01	MF1	0.44	2.07	0.21	YES	6.91
BrMYB68-BrMYB32	BrMYB68	A03	MF1	BrMYB32	A02	MF2	0.11	0.25	0.45	YES	0.84
BrMYB69-BrMYB33	BrMYB69	A03	MF1	BrMYB33	A02	MF2	0.06	0.23	0.25	YES	0.76
BrMYB93-BrMYB54	BrMYB93	A03	LF	BrMYB54	A02	MF1	0.1	0.39	0.26	YES	1.31
BrMYB92-BrMYB52	BrMYB92	A03	LF	BrMYB52	A02	MF1	0.14	0.38	0.37	YES	1.27
BrMYB57-BrMYB24	BrMYB57	A03	MF1	BrMYB24	A02	MF2	0.06	0.32	0.19	YES	1.07
BrMYB59-BrMYB25	BrMYB59	A03	MF1	BrMYB25	A02	MF2	0.08	0.8	0.1	YES	2.66
BrMYB66-BrMYB31	BrMYB66	A03	MF1	BrMYB31	A02	MF2	0.04	0.3	0.12	YES	1
BrMYB59-BrMYB51	BrMYB59	A03	MF1	BrMYB51	A02	MF1	0.43	3.27	0.13	YES	10.88
BrMYB60-BrMYB26	BrMYB60	A03	MF1	BrMYB26	A02	MF2	0.11	0.31	0.35	YES	1.02
BrMYB63-BrMYB28	BrMYB63	A03	MF1	BrMYB28	A02	MF2	0.08	0.44	0.19	YES	1.46
BrMYB58-BrMYB93	BrMYB58	A03	MF1	BrMYB93	A03	LF	0.31	1.24	0.25	YES	4.15
BrMYB78-BrMYB115	BrMYB78	A03	MF2	BrMYB115	A04	MF1	0.05	0.29	0.16	YES	0.96
BrMYB77-BrMYB121	BrMYB77	A03	MF2	BrMYB121	A05	LF	0.04	0.44	0.09	YES	1.47
BrMYB78-BrMYB120	BrMYB78	A03	MF2	BrMYB120	A05	LF	0.04	0.25	0.15	YES	0.84
BrMYB79-BrMYB117	BrMYB79	A03	MF2	BrMYB117	A05	LF	0.15	0.29	0.51	YES	0.97
BrMYB82-BrMYB135	BrMYB82	A03	MF2	BrMYB135	A05	LF	0.08	0.26	0.31	YES	0.87
BrMYB83-BrMYB132	BrMYB83	A03	MF2	BrMYB132	A05	LF	0.08	0.28	0.28	YES	0.95
BrMYB84-BrMYB131	BrMYB84	A03	MF2	BrMYB131	A05	LF	0.07	0.21	0.36	YES	0.68
BrMYB86-BrMYB128	BrMYB86	A03	MF2	BrMYB128	A05	LF	0.13	0.96	0.13	YES	3.2
BrMYB3R6-BrMYB3R7	BrMYB3R6	A03	MF2	BrMYB3R7	A05	LF	0.17	0.43	0.39	YES	1.45
BrMYB64-BrMYB133	BrMYB64	A03	MF1	BrMYB133	A05	LF	0.16	0.72	0.23	YES	2.4
BrMYB63-BrMYB134	BrMYB63	A03	MF1	BrMYB134	A05	LF	0.19	1.21	0.15	YES	4.03
BrMYB60-BrMYB148	BrMYB60	A03	MF1	BrMYB148	A06	LF	0.22	0.7	0.31	YES	2.33
BrMYB88-BrMYB166	BrMYB88	A03	MF2	BrMYB166	A07	LF	0.07	0.22	0.3	YES	0.72
BrMYB90-BrMYB165	BrMYB90	A03	MF2	BrMYB165	A07	LF	0.07	0.22	0.33	YES	0.75
BrMYB87-BrMYB191	BrMYB87	A03	MF2	BrMYB191	A08	MF1	0.4	1.45	0.28	YES	4.84
BrMYB96-BrMYB194	BrMYB96	A03	MF1	BrMYB194	A08	MF2	0.1	0.28	0.35	YES	0.95
BrMYB97-BrMYB195	BrMYB97	A03	MF1	BrMYB195	A08	MF2	0.1	0.34	0.28	YES	1.14
BrMYB94-BrMYB225	BrMYB94	A03	MF2	BrMYB225	A09	LF	0.12	0.68	0.17	YES	2.27
BrMYB92-BrMYB215	BrMYB92	A03	LF	BrMYB215	A09	MF2	0.09	0.38	0.24	YES	1.26
BrMYB3R6-BrMYB3R11	BrMYB3R6	A03	MF2	BrMYB3R11	A10	LF	0.38	1.92	0.2	YES	6.4
BrMYB57-BrMYB253	BrMYB57	A03	MF1	BrMYB253	A10	LF	0.07	0.55	0.13	YES	1.83
BrMYB58-BrMYB251	BrMYB58	A03	MF1	BrMYB251	A10	LF	0.01	0.33	0.03	YES	1.11
BrMYB59-BrMYB250	BrMYB59	A03	MF1	BrMYB250	A10	LF	0.09	0.65	0.14	YES	2.16
BrMYB60-BrMYB3R10	BrMYB60	A03	MF1	BrMYB3R10	A10	LF	0.09	0.16	0.55	YES	0.54
BrMYB3R5-BrMYB3R9	BrMYB3R5	A03	MF1	BrMYB3R9	A10	LF	0.1	0.48	0.21	YES	1.59
BrMYB61-BrMYB249	BrMYB61	A03	MF1	BrMYB249	A10	LF	0.08	0.23	0.34	YES	0.76
BrMYB62-BrMYB248	BrMYB62	A03	MF1	BrMYB248	A10	LF	0.08	0.3	0.26	YES	1.01
BrMYB63-BrMYB246	BrMYB63	A03	MF1	BrMYB246	A10	LF	0.07	0.43	0.16	YES	1.44
BrMYB64-BrMYB245	BrMYB64	A03	MF1	BrMYB245	A10	LF	0.08	0.37	0.21	YES	1.25
BrMYB87-BrMYB255	BrMYB87	A03	MF2	BrMYB255	Scaffold000164	-	0.07	0.16	0.46	YES	0.54
BrMYB114-BrMYB76	BrMYB114	A04	MF1	BrMYB76	A03	MF2	0.09	0.24	0.37	YES	0.79
BrMYB106-BrMYB211	BrMYB106	A04	LF	BrMYB211	A09	MF2	0.27	0.61	0.45	YES	2.04
BrMYB3R7-BrMYB3R2	BrMYB3R7	A05	LF	BrMYB3R2	A01	MF1	0.13	0.43	0.29	YES	1.45
BrMYB122-BrMYB76	BrMYB122	A05	LF	BrMYB76	A03	MF2	0.07	0.25	0.28	YES	0.83
BrMYB123-BrMYB75	BrMYB123	A05	LF	BrMYB75	A03	MF2	0.14	0.3	0.45	YES	1.01
BrMYB118-BrMYB116	BrMYB118	A05	LF	BrMYB116	A04	MF1	0.08	0.31	0.27	YES	1.03
BrMYB123-BrMYB113	BrMYB123	A05	LF	BrMYB113	A04	MF1	0.16	0.41	0.39	YES	1.38
BrMYB119-BrMYB228	BrMYB119	A05	LF	BrMYB228	A09	LF	0.3	1.86	0.16	YES	6.18
BrMYB126-BrMYB256	BrMYB126	A05	LF	BrMYB256	Scaffold000164	-	0.09	0.31	0.27	YES	1.05
BrMYB127-BrMYB254	BrMYB127	A05	LF	BrMYB254	Scaffold000164	-	0.06	0.44	0.13	YES	1.46
BrMYB150-BrMYB51	BrMYB150	A06	LF	BrMYB51	A02	MF1	0.27	0.45	0.6	YES	1.49
BrMYB151-BrMYB50	BrMYB151	A06	LF	BrMYB50	A02	MF1	0.12	0.33	0.36	YES	1.1
BrMYB152-BrMYB49	BrMYB152	A06	LF	BrMYB49	A02	MF1	0.1	0.35	0.28	YES	1.18
BrMYB147-BrMYB55	BrMYB147	A06	LF	BrMYB55	A02	MF1	0.06	0.25	0.23	YES	0.83
BrMYB146-BrMYB31	BrMYB146	A06	LF	BrMYB31	A02	MF2	0.13	0.93	0.14	YES	3.12
BrMYB148-BrMYB26	BrMYB148	A06	LF	BrMYB26	A02	MF2	0.19	0.9	0.21	YES	2.99
BrMYB156-BrMYB46	BrMYB156	A06	LF	BrMYB46	A02	MF1	0.04	0.24	0.15	YES	0.82
BrMYB140-BrMYB44	BrMYB140	A06	LF	BrMYB44	A02	MF1	0.14	0.73	0.19	YES	2.43
BrMYB160-BrMYB45	BrMYB160	A06	LF	BrMYB45	A02	MF1	0.19	0.74	0.25	YES	2.48
BrMYB160-BrMYB108	BrMYB160	A06	LF	BrMYB108	A04	LF	0.59	2.63	0.22	YES	8.78
BrMYB158-BrMYB107	BrMYB158	A06	LF	BrMYB107	A04	LF	0.31	2.6	0.12	YES	8.68
BrMYB140-BrMYB161	BrMYB140	A06	LF	BrMYB161	A06	LF	0.16	0.88	0.18	YES	2.92
BrMYB141-BrMYB190	BrMYB141	A06	LF	BrMYB190	A07	LF	0.25	0.86	0.29	YES	2.87
BrMYB137-BrMYB207	BrMYB137	A06	LF	BrMYB207	A08	MF1	0.08	0.3	0.28	YES	1
BrMYB139-BrMYB205	BrMYB139	A06	LF	BrMYB205	A08	MF1	0.03	0.34	0.08	YES	1.13
BrMYB138-BrMYB206	BrMYB138	A06	LF	BrMYB206	A08	MF1	0.07	0.33	0.23	YES	1.08
BrMYB159-BrMYB237	BrMYB159	A06	LF	BrMYB237	A09	MF2	0.06	0.33	0.17	YES	1.1
BrMYB151-BrMYB214	BrMYB151	A06	LF	BrMYB214	A09	MF2	0.09	0.27	0.34	YES	0.9
BrMYB147-BrMYB217	BrMYB147	A06	LF	BrMYB217	A09	MF2	0.07	0.22	0.32	YES	0.74
BrMYB138-BrMYB235	BrMYB138	A06	LF	BrMYB235	A09	MF2	0.11	0.4	0.28	YES	1.32
BrMYB149-BrMYB218	BrMYB149	A06	LF	BrMYB218	A09	MF2	0.07	0.31	0.23	YES	1.05
BrMYB158-BrMYB209	BrMYB158	A06	LF	BrMYB209	A09	MF2	0.1	0.45	0.23	YES	1.51
BrMYB156-BrMYB210	BrMYB156	A06	LF	BrMYB210	A09	MF2	0.06	0.36	0.18	YES	1.2
BrMYB169-BrMYB11	BrMYB169	A07	LF	BrMYB11	A01	MF1	0.3	1.9	0.16	YES	6.34
BrMYB176-BrMYB42	BrMYB176	A07	MF2	BrMYB42	A02	MF1	0.11	0.31	0.35	YES	1.04
BrMYB177-BrMYB41	BrMYB177	A07	MF2	BrMYB41	A02	MF1	0.17	0.41	0.42	YES	1.36
BrMYB181-BrMYB37	BrMYB181	A07	LF	BrMYB37	A02	MF1	0.11	0.35	0.31	YES	1.17
BrMYB167-BrMYB38	BrMYB167	A07	MF2	BrMYB38	A02	MF1	0.33	1.59	0.21	YES	5.3
BrMYB182-BrMYB38	BrMYB182	A07	LF	BrMYB38	A02	MF1	0.08	0.35	0.22	YES	1.16
BrMYB179-BrMYB38	BrMYB179	A07	MF2	BrMYB38	A02	MF1	0.07	0.34	0.21	YES	1.14
BrMYB169-BrMYB104	BrMYB169	A07	LF	BrMYB104	A03	MF1	0.29	3.46	0.08	YES	11.53
BrMYB173-BrMYB105	BrMYB173	A07	MF2	BrMYB105	A04	MF1	0.06	0.27	0.22	YES	0.89
BrMYB169-BrMYB111	BrMYB169	A07	LF	BrMYB111	A04	MF1	0.28	3.42	0.08	YES	11.38
BrMYB164-BrMYB129	BrMYB164	A07	LF	BrMYB129	A05	LF	0.14	0.48	0.29	YES	1.59
BrMYB174-BrMYB141	BrMYB174	A07	MF2	BrMYB141	A06	LF	0.26	1.17	0.23	YES	3.91
BrMYB185-BrMYB142	BrMYB185	A07	LF	BrMYB142	A06	LF	0.29	1.29	0.22	YES	4.29
BrMYB177-BrMYB143	BrMYB177	A07	MF2	BrMYB143	A06	LF	0.34	2.68	0.13	YES	8.95
BrMYB178-BrMYB183	BrMYB178	A07	MF2	BrMYB183	A07	LF	0.1	0.23	0.45	YES	0.75
BrMYB182-BrMYB167	BrMYB182	A07	LF	BrMYB167	A07	MF2	0.28	1.56	0.18	YES	5.2
BrMYB176-BrMYB188	BrMYB176	A07	MF2	BrMYB188	A07	LF	0.08	0.25	0.33	YES	0.85
BrMYB177-BrMYB186	BrMYB177	A07	MF2	BrMYB186	A07	LF	0.08	0.49	0.17	YES	1.62
BrMYB174-BrMYB190	BrMYB174	A07	MF2	BrMYB190	A07	LF	0.11	0.46	0.25	YES	1.53
BrMYB179-BrMYB167	BrMYB179	A07	MF2	BrMYB167	A07	MF2	0.27	1.48	0.18	YES	4.94
BrMYB186-BrMYB202	BrMYB186	A07	LF	BrMYB202	A08	MF1	0.37	1.56	0.23	YES	5.21
BrMYB178-BrMYB201	BrMYB178	A07	MF2	BrMYB201	A08	MF1	0.32	1.76	0.18	YES	5.88
BrMYB172-BrMYB229	BrMYB172	A07	MF2	BrMYB229	A09	LF	0.1	0.54	0.19	YES	1.79
BrMYB168-BrMYB224	BrMYB168	A07	MF2	BrMYB224	A09	LF	0.07	0.3	0.25	YES	1
BrMYB187-BrMYB231	BrMYB187	A07	LF	BrMYB231	A09	MF2	0.45	1.94	0.23	YES	6.48
BrMYB173-BrMYB230	BrMYB173	A07	MF2	BrMYB230	A09	LF	0.06	0.41	0.15	YES	1.35
BrMYB174-BrMYB234	BrMYB174	A07	MF2	BrMYB234	A09	MF2	0.24	1.21	0.2	YES	4.05
BrMYB182-BrMYB3R8	BrMYB182	A07	LF	BrMYB3R8	A09	LF	0.26	1.43	0.18	YES	4.78
BrMYB184-BrMYB233	BrMYB184	A07	LF	BrMYB233	A09	MF2	0.23	1.43	0.16	YES	4.77
BrMYB185-BrMYB232	BrMYB185	A07	LF	BrMYB232	A09	MF2	0.28	1.09	0.25	YES	3.64
BrMYB177-BrMYB231	BrMYB177	A07	MF2	BrMYB231	A09	MF2	0.37	2.82	0.13	YES	9.4
BrMYB169-BrMYB225	BrMYB169	A07	LF	BrMYB225	A09	LF	0.27	1.54	0.18	YES	5.15
BrMYB199-BrMYB2	BrMYB199	A08	MF2	BrMYB2	A01	LF	0.06	0.41	0.15	YES	1.38
BrMYB199-BrMYB104	BrMYB199	A08	MF2	BrMYB104	A03	MF1	0.11	0.33	0.33	YES	1.1
BrMYB192-BrMYB136	BrMYB192	A08	MF1	BrMYB136	A06	LF	0.07	0.36	0.19	YES	1.22
BrMYB203-BrMYB142	BrMYB203	A08	MF1	BrMYB142	A06	LF	0.11	0.3	0.36	YES	1
BrMYB200-BrMYB163	BrMYB200	A08	MF2	BrMYB163	A06	MF1	0.06	0.28	0.23	YES	0.92
BrMYB204-BrMYB233	BrMYB204	A08	MF1	BrMYB233	A09	MF2	0.1	0.44	0.23	YES	1.47
BrMYB3R8-BrMYB38	BrMYB3R8	A09	LF	BrMYB38	A02	MF1	0.3	1.2	0.25	YES	4.01
BrMYB226-BrMYB95	BrMYB226	A09	LF	BrMYB95	A03	MF2	0.1	0.24	0.44	YES	0.8
BrMYB222-BrMYB98	BrMYB222	A09	LF	BrMYB98	A03	MF1	0.22	3.47	0.06	YES	11.57
BrMYB230-BrMYB105	BrMYB230	A09	LF	BrMYB105	A04	MF1	0.08	0.39	0.21	YES	1.31
BrMYB213-BrMYB154	BrMYB213	A09	MF2	BrMYB154	A06	LF	0.1	0.23	0.44	YES	0.75
BrMYB227-BrMYB171	BrMYB227	A09	LF	BrMYB171	A07	MF2	0.09	0.2	0.45	YES	0.68
BrMYB234-BrMYB190	BrMYB234	A09	MF2	BrMYB190	A07	LF	0.24	0.95	0.25	YES	3.16
BrMYB220-BrMYB219	BrMYB220	A09	MF1	BrMYB219	A09	MF1	0.07	0.33	0.2	YES	1.11
BrMYB252-BrMYB15	BrMYB252	A10	LF	BrMYB15	A01	MF1	0.16	0.95	0.16	YES	3.16
BrMYB253-BrMYB3R1	BrMYB253	A10	LF	BrMYB3R1	A01	MF1	0.53	1.85	0.28	YES	6.18
BrMYB3R11-BrMYB3R2	BrMYB3R11	A10	LF	BrMYB3R2	A01	MF1	0.34	2.77	0.12	YES	9.23
BrMYB239-BrMYB34	BrMYB239	A10	LF	BrMYB34	A02	MF2	0.03	0.31	0.09	YES	1.03
BrMYB247-BrMYB27	BrMYB247	A10	LF	BrMYB27	A02	MF2	0.04	0.3	0.13	YES	1.01
BrMYB244-BrMYB29	BrMYB244	A10	LF	BrMYB29	A02	MF2	0.11	0.26	0.43	YES	0.88
BrMYB242-BrMYB30	BrMYB242	A10	LF	BrMYB30	A02	MF2	0.07	0.2	0.34	YES	0.67
BrMYB238-BrMYB35	BrMYB238	A10	LF	BrMYB35	A02	MF2	0.12	0.32	0.38	YES	1.07
BrMYB246-BrMYB28	BrMYB246	A10	LF	BrMYB28	A02	MF2	0.08	0.46	0.17	YES	1.52
BrMYB3R10-BrMYB26	BrMYB3R10	A10	LF	BrMYB26	A02	MF2	0.08	0.35	0.22	YES	1.18
BrMYB250-BrMYB51	BrMYB250	A10	LF	BrMYB51	A02	MF1	0.48	1.54	0.31	YES	5.15
BrMYB241-BrMYB31	BrMYB241	A10	LF	BrMYB31	A02	MF2	0.04	0.29	0.15	YES	0.95
BrMYB244-BrMYB65	BrMYB244	A10	LF	BrMYB65	A03	MF1	0.13	0.29	0.44	YES	0.97
BrMYB241-BrMYB66	BrMYB241	A10	LF	BrMYB66	A03	MF1	0.04	0.37	0.1	YES	1.23
BrMYB240-BrMYB67	BrMYB240	A10	LF	BrMYB67	A03	MF1	0.05	0.45	0.11	YES	1.51
BrMYB238-BrMYB70	BrMYB238	A10	LF	BrMYB70	A03	MF1	0.08	0.46	0.18	YES	1.52
BrMYB236-BrMYB74	BrMYB236	A10	LF	BrMYB74	A03	MF2	0.32	1.3	0.25	YES	4.34
BrMYB236-BrMYB112	BrMYB236	A10	LF	BrMYB112	A04	MF1	0.25	1.34	0.19	YES	4.46
BrMYB242-BrMYB122	BrMYB242	A10	LF	BrMYB122	A05	LF	0.42	2.51	0.17	YES	8.37
BrMYB245-BrMYB133	BrMYB245	A10	LF	BrMYB133	A05	LF	0.17	0.88	0.19	YES	2.95
BrMYB252-BrMYB130	BrMYB252	A10	LF	BrMYB130	A05	LF	0.17	1.18	0.14	YES	3.94
BrMYB252-BrMYB134	BrMYB252	A10	LF	BrMYB134	A05	LF	0.19	1.09	0.18	YES	3.65
BrMYB241-BrMYB146	BrMYB241	A10	LF	BrMYB146	A06	LF	0.14	0.8	0.17	YES	2.68

Abbreviations: LF: Less Fractioned subgenome; MFs (MF1 and MF2), More Fractioned subgenomes; MYA, million year ago.

It is likely that after duplication, a series of synonymous and/or non-synonymous mutations in their ORFs generated new functions for the *BrMYBs* during evolution. Therefore, we calculated the synonymous (Ks) and nonsynonymous substitutions (Ka) per site between duplicated gene pairs to estimate the selection types and divergence timing. The calculation results for the 185 duplicated pairs are listed in Table 1. All duplicated *R2R3-MYB* gene pairs had a Ka/Ks ratio < 1, representing purifying selection (Table 1). All of the duplicated genes were found to be segmentally duplicated according to the classify method constructed previously [37], which are located on duplicated segments on 10 chromosomes and 3 subgenomes in Chinese cabbage. Among them, *R2R3-MYB* genes containing subgenome LF have one or more duplicated genes in other subgenomes, suggesting that all the BrMYB genes have been retained in Chinese cabbage after genome triplications. In previous reports, estimations of cruciferous plant evolutionary timescales were based on the synonymous substitution rate [38]. The divergence times of the duplicated *R2R3-MYB*s were also calculated as described in the “Methods”. The divergence time ranged from 0.54 (for *BrMYB60-BrMYB3R10* and *BrMYB87-BrMYB255*) to 12.83 (for *BrMYB3-BrMYB163*) million years (Table 1). This indicated that the duplication events for most R2R3-MYBs in Chinese cabbage occurred after the genome triplication event (i.e., 5–9 MYA) [39]. Their duplication seemed to be inconsistent with the whole-genome duplication, it might be caused by the genome partly deletion, and the lacking degree of the three subgenomes were distinct, which further leaded to the delay of the calculated divergence times. In contrast, the divergence times of *BrMYB3R11-BrMYB3R2*, *BrMYB177-BrMYB231*, *BrMYB59-BrMYB51*, *BrMYB39-BrMYB233*, *BrMYB169-BrMYB111*, *BrMYB169-BrMYB104*, *BrMYB222-BrMYB98* and *BrMYB3-BrMYB163* were earlier than the triplication event.

### Phylogenetic analysis and conserved motif identification of the R2R3-MYB family in Chinese cabbage

**Figure 6:**
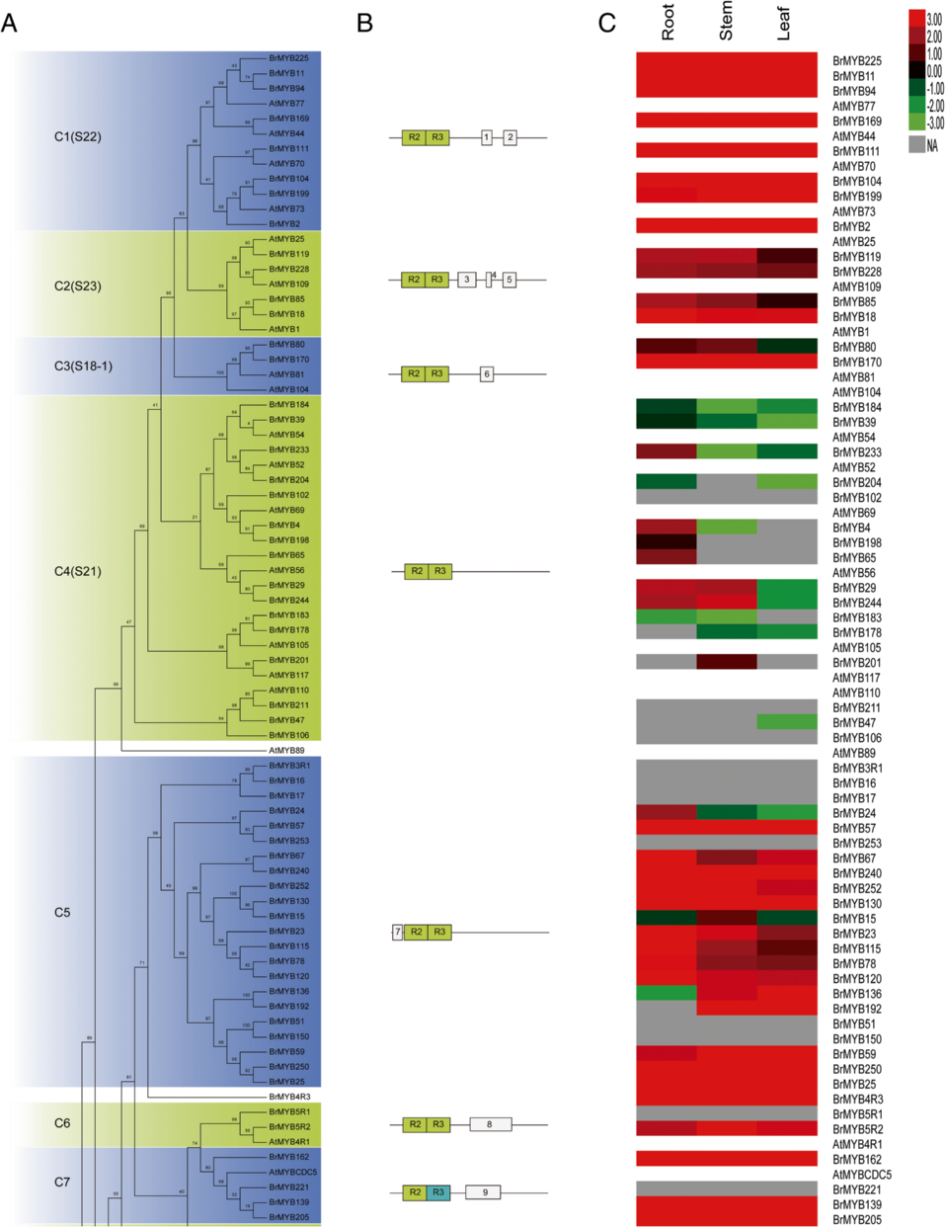
**Part of the phylogenetic relationships and subgroup designations of MYB proteins from Chinese cabbage and** ***Arabidopsis*** **. (A)** Maximum-Likelihood tree representing the relationships among 274 MYB proteins from Chinese cabbage and 132 from *Arabidopsis*, including seven and 17 3R- and atypical MYBs from *Arabidopsis* and Chinese cabbage, respectively. The proteins are clustered into 45 subgroups, each designated with a subgroup number (e.g., C1). The numbers beside the branches represent bootstrap support values (>50%) from 1000 replications. Five proteins did not fit well into clusters. **(B)** Architecture of conserved protein motifs in the 45 subfamilies. The motifs on the right were analyzed using MEME and are represented as boxes. **(C)** Expression patterns of MYB genes in Chinese cabbage in different tissues (root, leaf and stem). The Illumina RNA-seq data were reanalyzed, and the RPKM values were log 2 transformed and a heat map was generated using the Cluster 3.0 software. The bar on the left represents log 2 transformed values from low to high expression. NA represents no available data.

To evaluate the evolutionary relationships within the R2R3-MYB gene family, we performed a combined phylogenetic analysis of *Arabidopsis* and Chinese cabbage R2R3-MYB proteins (including 7 and 17 members with more than two MYB domains, respectively) to obtain a Maximum Likelihood (ML) tree using MEGA 5 (1000 bootstrap replicates, Figure 6A and Additional file 4: Figure S3). Because of the large number of taxa and relatively low support values for informative characters, we used NJ analysis to support our subgroup designations (Additional file 5: Figure S4). The tree topologies derived from the ML and NJ analyses were basically identical, which indicated that the two methods were in strong agreement. Five sequences did not belong to any of the subfamilies (Figure 6A and Additional file 6: Table S3). The sequence similarity and phylogenetic tree topology allowed us to divide the genes into 45 subfamilies, which ranged in size from 2 to 23 *MYB*s (Figure 6A and Additional file 6: Table S3 and Additional file 4: Figure S3). In our subfamily classification of *MYB* genes, we also referred to the classification model of *Arabidopsis R2R3-MYB* genes constructed by Stracke *et al.* and Dubos *et al.* [4,27]. In *Arabidopsis*, 90 of the 126 *R2R3-MYB*s had been divided into 25 subfamilies (S1–25), so we labeled the previously defined clades in the trees shown in Figure 6A and Additional file 4: Figure S3 to compare our results with these studies. Most of the large subgroups (e.g., C4, C10 and C14) were supported by previous studies while some small ones (e.g., C6–9) were not. The unequal distribution of *R2R3-MYBs* between Chinese cabbage and *Arabidopsis* further supported the existence of the *B. rapa* whole genome triplication event (Table 1 and Figure 6A). In most subgroups defined in our ML tree, there were more *R2R3-MYBs* in Chinese cabbage than *Arabidopsis*; by contrast, the C13 subgroup included an equal number of *MYBs* from *Arabidopsis* and Chinese cabbage. These findings indicated that the *R2R3-MYBs* in Chinese cabbage experienced duplications after the divergence of Chinese cabbage and *Arabidopsis*. Notably, subgroups C5 and C22 contained 22 and 5 *R2R3-BrMYBs* but no *R2R3-AtMYBs*, which suggested that the members of these subfamilies might have specialized roles that were either lost in *Arabidopsis* or acquired in the Chinese cabbage lineage after divergence from the last common ancestor with *Arabidopsis* (Figure 6A and Additional file 6: Table S3). To determine whether this *Arabidopsis* ortholog gene loss phenomenon was unique to dicots or also extended to monocots, we constructed a ML phylogenetic tree of *R2R3-MYB*s from *Arabidopsis*, Chinese cabbage and rice (Additional file 7: Figure S5). The tree topology showed there were also ancestral duplication and gene loss events in rice *R2R3-MYB*s. Taking previous studies on poplar into consideration [23], we suggest that the ancestral duplication of *R2R3-MYB* genes might extend to various types of land plant species.

Genes from the same subfamily sharing the same motifs are likely to share similar functions [4]. Since our classification was based on the *Arabidopsis* model, which was grouped according to the functions of the AtMYBs, we could further explore the common motifs and potential functions of each R2R3-BrMYB group. Using MEME, we searched for conserved motifs outside of the MYB domains of each group. Thirty-one of the 45 classified subfamilies shared one or more motifs outside of the MYB domains, which provided further support for the subfamily definitions. We identified 45 conserved motifs in the C-terminal regions and two motifs located before the MYB domains (Figure 6B and Table 2), ranging in size from 8 to 110 amino acids. This was consistent with previous hypothesis that MYBs with similar protein structures were clustered into the same conserved subfamily [4]. Most of these conserved motifs were novel, but some had been characterized with different functions [27]. For instance, motif 8, which was conserved in C6 members, was characterized as involved in apoptotic signaling and has SPT5 protein-binding characteristics according to SMART analysis. These results suggest that these genes probably participate in cell death processes regulated by SPT5-mediated transcriptional elongation [40]. In many plants, R2R3-MYB (such as AtMYB95), bHLH and WD-repeat proteins regulate the anthocyanin biosynthesis pathway [41]. The companions of AtMYB95 in C43 contained motif 46, which is known to participate in WD-repeat interactions. In *Arabidopsis*, AtMYBCDC5 (AtMYB125) is distantly related to typical R2R3-MYB proteins [4,27]. AtMYBCDC5 contains an R3-repeat in its MYB domain that shows low homology to typical R3-repeats in R2R3-MYBs (Figure 5B, marked with a blue box), and a very long C-terminal region. In Chinese cabbage, four genes (*BrMYB246*, *BrMYB286*, *BrMYB364* and *BrMYB389*) were highly homologous to AtMYBCDC5 (Figure 6A, subfamily C7). A MEME search identified a conserved 80 amino acid motif in the C-terminal region of this subfamily (Figure 6B and Additional file 6: Table S3) that might interact with histone deacetylase (HDAC) proteins, raising the possibility that these subfamily members might be involved in HDAC-mediated transcription inhibition [42].

**Table 2:** **Conserved motif analysis of 273 R2R3-BrMYB proteins**

**Clade**	**Motif**	**Width**	**E-value**	**Multilevel consensus sequence**
1	1	21	1.30E-71	EDP[PA]T[SY]LSLSL[PS][WGL][ANP][ND]ES[EV][TS][ES]N
	2	33	2.00E-212	GEFMTVVQEMI[KR][TA]EVRSYMA[ED][LM]QR[GN][NS][GV]GGG[GV][GS]G
2	3	41	4.50E-88	[MPV][HV]PCE[GQ][PN][LK][FIV]Q[AS][ACS][KR][PQ]D[SA][LA][AM][GL][KR][FL]L[QE][SG][LA][CY][SY]E[PR][FN][VI]P [SQ]KCGHGC[CS][SNT]
	4	14	4.00E-36	S[VL]LGPEFVDY[EL][ED]P[PS]
	5	31	3.70E-57	[SDN][QY]EL[AI][SA]IAT[DE][LI][NS][NS][IL]AW[IL][RK]SGL[ED][NS][SA]SVRE[AM]E[QDE]
3	6	34	4.40E-34	[RG][KN]R[VFP][RM]EPET[ADT]F[PL][CDFY][TP]GG[YS][AT][MAT][ND]EQ[SN][PAG][QRT]L[WL][NC][YNS]P[FY]VE[SN]
5	7	21	1.70E-182	W[TS]RE[ED][ND][KI]AFE[NR]ALA[IV][YF][DP][DE][DE][ST][PE]
6	8	110	6.40E-149	ADVEA[QH]LR[KR]QDVARNKIA[EQ]R[RQ]DAPAAILQANK[LM]NDPE[AV]VRKRSKLMLPPPQISDHELEEIAKMGYASDLL AENEEL[TM]EGSAATRALLANYSQTPRQGMTP[ML]RTPQRTPAGKGDAIMMEAENLARLRDSQTPLLGG[DE]NPELHPSDFTG VTPRKKEIQTPNPMLTPSMTP
7	9	80	6.30E-67	GGA[GS]LTPR[IL]GLTPSR[DE]GSSF[AS][MV]TP[RK]GTPFRDELHINEDMDMHE[SN]AKLERQRREEAR[RM]SLRSGLTGLP[Q L]P[RK]NEYQIVAQ[PA]P
8	10	101	4.60E-96	SVF[ML]SEL[VM]ECCRE[LV]EEGHRAWA[ED]HKKEAAWRLRRLELQLESEKT[SC]RQREK[MT]EEIE[AT]KMKALREEQK[M N]AMEKIEGEYREQLVGLRRDAEAK[DE]QKLADQW[TS]S[KR]HIRLTKFLEQ[HQ]MGCR
9	11	72	9.30E-166	[NG][EQH][EG][VM]FLKKDD[PS]K[VA]T[ACN]LMQQAELLSSLA[QH]KVN[AS][DE]NT[ED]QSMENAWKVLQDF[LF]NK[SG] KEND[LI][FL]RYG[ILF]P[DE][IM]DF[QK][LI][ED]E
	12	37	3.30E-66	F[KR]DL[VI][EDG]DLRS[SGT][NY]E[DA][SN][QD][ALPSV]S[WLY]RQPDLHDSPASS[ED][YN]SSGS
	13	20	3.60E-15	T[ITV][MI][PLTV][HD][PQS]SGD[KQ]TQ[QP][FLPQS][MLPS][SAMP][DGS][STP][QDP][TQ]
	14	33	5.70E-66	F[NS]SP[VI]QVTPLFRSLA[AD]GIPSPQFSES[EV][RS][SIN][FH][LV][LT][KN]
	15	21	1.40E-31	PCPSAN[PL]S[QK]PPPCKRVLL[DH]SL
10	16	16	1.20E-102	[CK][RS][SAP][NK][APY][PK]R[PN][SN][IL]L[QE][DN]YI[RK]S[IVL]TN[NG]N[EL]SxS[ST]V[EP]
14	17	29	2.50E-58	ATSS[CS][VAI]T[TS]SN[DNS][QP][FA][MEL][TI][YT][SD][YD][NDV]N[GN]N[VMN][GNV][NQ][GNQ][FT]G[VY]
15	18	33	2.30E-55	RA[EH][EQ]ES[DE]EDEV[KD]KWFKHLESELGLEE[DN]D[NS]QQ[QH]Q
16	19	39	4.80E-22	MES[DG]KE[TA][NC]G[GV][IV][CFG][EG][RT]E[SR]FGVM[KN]SPYENRI[SI]DWIS[EK][IS][SD][TA][DN][QI]
17	20	21	4.10E-34	[DN][GN][SM][SD][SC]S[ST][ST][FM][MS][PQ]DL[TM][TK]V[PS][HQ]F[MI]D
	21	21	1.50E-93	[AY][YE]EDVTQDPMWN[MV]DDIWQF[RE]E
18	22	51	2.60E-50	[HS][HQ]SSEIND[QH][AV]ASTS[ANS]HNVFC[TA]QDQAM[ED]TYSPT[TP]TSYQHTNM[ED]FNYGN[YF]SA[AP]
20	23	29	2.00E-94	D[GD]YYSMDDIWREID[QH]S[GA][AV]NIIKPVKDIYY
	24	28	2.70E-70	[FY]P[PN]LASP[TA]WESSL[ED]SIWNMDAD[EK]SK[MI]SS
21	25	27	1.30E-66	V[PA][VA][TP][SI][SP][DE][AH][NS][MV][NI][ED][ED][GN][AN][IL]W[DG][GS]LW[NS]LD[DL][ED][DG]
24	26	15	1.10E-43	WV[HL][DE]D[DE]FELS[ST]LT[MN]M
26	27	40	1.40E-35	[IP][TQ][TN]NN[PL]F[PT][TA][PG][HN]M[IF]SH[PS][CF][NI][DE]DFTP[YC]VDG[LI]YGVN[TA]G[LV]QGELY
	28	29	7.40E-22	PPLEC[EQ]EGDWY[KN]A[DEN]IN[NS]H[LV][DA][ED][LMV][NK]TNG[AS]GN
	29	24	2.90E-24	[VM]EE[CFY]WDLDQLM[NS]TEVPSF[YH]FNF[KN][QF]
27	30	41	6.80E-27	[TH][SH][HT][KH][DP]N[KD][LV][KQ][SW]PS[LQ][PT][DT][LI]P[AS][QS]T[IV][IS]P[IF][NQ]ET[LM][SQ][DS][LY][DL]DG[E N][KN][LM][NI][VP]F[VW]
32	31	16	7.10E-50	[MT]F[LM]DY[CN][QL][DE][FY]GV[HE]D[FV][GP]F
33	32	81	7.80E-71	[ED]WF[LI]P[PA]SEN[TI]N[AGV]I[AP]C[AST]TSNNLN[LV][EQ][AV]LD[PL]CF[NS]SK[NT][ML]CHSESFKVGN[IMV][FLM]G [MI]EN[AGS]SWE[IT]ENPKIGDWDLD[GS]LIDNNSSFPFLDFQV
34	33	21	1.30E-20	EDFGFCYDDKFSSFLN[SA][LV]IND
35	34	21	4.20E-26	[PR][PL][PQ][AEPT]KRR[LP]GRTSRS[AT]MKPK[TFI][HI]
	35	21	4.70E-27	[HQ][QKV][NV][NI][DEN][AT][FLMS][TA]D[ED]F[IV]DWD[CF]VW[QR][EQ]G
	36	48	7.20E-30	[DH]EKE[AGN][SP]D[SP][MV]VSWLL[DN]G[DE]DEATIG[KNQ]SNCE[NK][FS]GEPLDHD[ED]E[NS]ALVAWLLS
36	37	29	2.90E-197	[TR][TN][QH][EY][TP][ST][GTS][TV]YASS[TA][ED]NI[AS][KR]LL[QK][GND][WF][MTV][KS][DS][ST][PS][KS]
37	38	18	1.70E-23	[PT][GNP][LFNS][DEN][DEV][YF][ND]EW[LF][NSI][FY][FILM]DNQ[TA][YCFL]
38	39	32	1.40E-93	[KD][DQ][DS][CAM][MFT]S[FY]E[DN][FIV][GSL]A[DL][IV]D[ED]SFW[SN][ED][VAT][VL][SY][SVM][DQ][DNC]
	40	36	1.00E-96	[EG][ILM][KLQ][QD][ER][NF][QSW][KEQ][LR][GS][SL][YDV][NGS]N[ES][KM][LGIV][YF][ND][DSH][DE]M[ED]FW[FY]DV [LF][TAL][RS][ST][GRS][GR][EI][QEI]
40	41	29	8.40E-70	[DA][AL][TE][STD][LV][AL]K[AL]QL[LI]H[KNS]M[IL]Q[VI][LI][SNT][PTN][NK][NA][NIT][PNST][NST][ISP][SN][SD][FSI]
41	42	29	5.80E-58	Q[EDT][QS][TAQ][IFL][LS][KNS]LQ[TG]E[MA]A[KQ]L[QA]L[FL]QYLLQ[PM][PS][SNP][MS]S[NAM]
	43	24	4.30E-54	A[ST]SS[SH][SG][QY][EG][SV][GA][AE][ST]AS[AV][ADY]WPDH[LC][LF]D[DE]
42	44	32	4.40E-200	[QL][SA][KP][NA][AS][AP]T[LT][SR]HMAQWESARLEAEARL[AS]RES[KM]L[LF]
	45	20	1.50E-79	[QD][QL][QL]L[ED][SF]P[TI]S[TD][VD][DST][FM]S[EF][LMN][EKL]ENI
43	46	41	1.40E-124	[TP][ST][SP][ST][ST][STE][TS][SH][SF]S[SF]S[SP][TS][GS]S[AV][RC]LLNKLA[AT]GISSR[KQ]H[DAG]LDRIK[NT][VI][IL]
44	47	8	6.40E-100	M[VS]R[TK]PCC[KV]

Significant motifs (e-value < e-100) of more than 10 amino acid length were predicted by MEME analysis. Motif ID, consensus sequences, width (amino acids), and number of R2R3-BrMYB proteins containing the motif and e-value of each predicted motif is given.

### Expression profiling of *R2R3-MYB* genes in Chinese cabbage

Previously developed RNA-seq web-based tools, including tissue-specific gene expression data, allowed us to analyze the transcriptome in Chinese cabbage [39]. Then, different transcript patterns were identified for the 273 *R2R3-MYB* genes (including 3R and atypical *MYB*s) using BRAD data, and gene expression levels were calculated in RPKM units (Additional file 8: Table S2). Consequently, we obtained expression information for each subfamily and compared the expression profiles of MYB transcription factor subfamilies of Chinese cabbage in different tissues (root, stem and leaf). We subsequently summarized these expression profiles against the phylogenic tree (Figure 6C).

As with many genes encoding transcription factors, many of the *R2R3-BrMYB* genes had low transcript levels according to the RNA-seq analysis. However, different transcript abundance patterns were identified in the RNA-seq dataset for the *R2R3-BrMYB* genes. The RPKM values of the *R2R3-MYB* genes are shown in Additional file 8: Table S2. Among the 273 genes, 234 were expressed in at least one tissue, while the remaining 39 members either had no expression or their expression profiles could not be found in the RNA-seq database. Nearly 120 of the 234 genes (~51%) were expressed at relatively low levels in all three tissues. For example, the expression of 29 *R2R3-BrMYB*s (~12%) in the roots, 66 (~28%) in the stem, and 61 (~26%) in the leaves was downregulated (Figure 6C). However, 35 (~15%) R2R3-MYBs showed high transcript levels in all three tissues, indicating that they might be indispensable in maintaining normal growth and metabolic processes of Chinese cabbage. In contrast, 101 (~43%) of the 234 genes had marked peaks in transcript levels in only one tissue, including 73 in the roots, 18 in the stem and 10 in the leaves, which suggests that these R2R3-BrMYB proteins act as regulators limited to discrete tissues or organs. For instance, 12 of the *R2R3-BrMYB* members with the most abundant expression in roots encode proteins in subfamily C30 (Figure 6A). The C30 (S14) subfamily containing *Arabidopsis MYB*s (*AtMYB37*, *38*, *68* and *84*) has been reported to function in the regulation of root development and axillary meristems [43,44]. This suggests that *R2R3-BrMYB* members of this subfamily that are expressed in Chinese cabbage roots may have a similar role in determining root architecture. *BrMYB246* had the highest transcript abundance in leaves, and its homologous gene *AtMYB16* has been shown to regulate cuticle formation in trichomes and induce over-accumulation of waxy substances on leaves [45]; thus, we could deduce from the peak expression of *BrMYB246* in leaves that it probably be involved in waxy substance formation to protect the leaves of Chinese cabbage. In addition, some of the *R2R3-BrMYB*s exhibited tissue-specific expression. For example, consistent with its *Arabidopsis* homolog *AtMYB72*, *BrMYB191* was only expressed in the root, indicating a role in rhizobacteria-induced systemic resistance such as *AtMYB72* performs in *Arabidopsis* roots [46]. However, in contrast with the expression profile of *AtMYB110*, which was shown to function in seed size regulation [47], its Chinese cabbage homolog *BrMYB211* was only expressed in the stem. Overall, although the functions of most *R2R3-BrMYB* genes are unknown, our phylogenetic and expression profiling analyses provide a foundation for further research on *R2R3-BrMYB* gene functions.

### *R2R3-BrMYB*s involved in abiotic stresses and signal transduction

**Figure 7:**
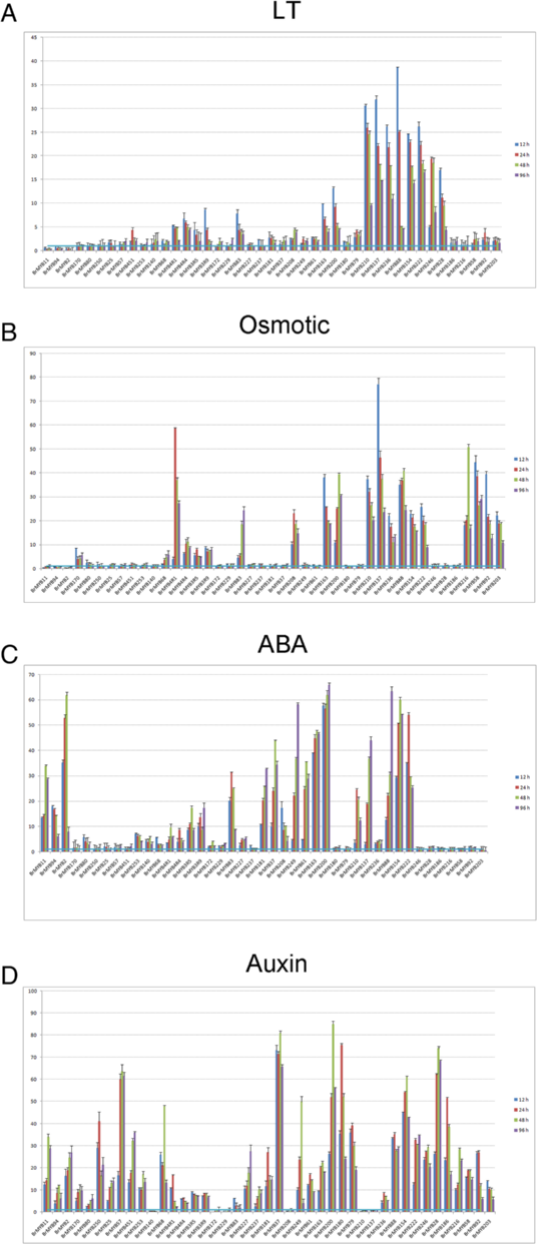
**Expression patterns of** ***R2R3-MYB*** **genes under abiotic stress and hormone treatments.** Four-leaf-stage Chinese cabbage plants were given various treatments, including **(A)** cold, **(B)** osmotic stress, **(C)** ABA and **(D)** auxin, under a continuous time course (0, 12, 24, 48, and 96 h). qPCR data were normalized using *BrGAPDH*. Results are the means ± standard deviation (SD) of three independent experiments.

R2R3-MYB proteins that have been characterized mainly participate in plant-specific processes, such as primary and secondary metabolism, cell identity, developmental regulation and stress responses [4,24]. In nature, plants suffer various biotic and abiotic stresses throughout their growth and development. Some R2R3-MYBs, such as AtMYB2, AtMYB6 and AtMYB30 are involved in responses to these stresses [48]. We selected forty-three *R2R3-BrMYB*s that had relatively remarkable expression in the expression profiles above for qPCR analysis of their responses to abiotic stresses (cold and osmotic stress) and signaling hormones (ABA and auxin), to explore whether these *BrMYB* genes had significant performance in response to exogenous stressors. The overall expression trends of these selected genes in response to cold stress were similar under osmotic stress, and more than half of the selected *R2R3-BrMYB* genes were differentially expressed under at least one stressor (cold and/or osmotic stress) (Figure 7A,B). Most of the *R2R3-BrMYB* genes up-regulated by cold or osmotic stress reached their peak expression at about 12 h after treatment, indicating that their stress response might be rapidly regulated. By contrast, some selected genes such as *BrMYB80*, *BrMYB170*, and *BrMYB250* were continuously expressed (i.e., at 0, 12, 24, 48 and 96 h) under different abiotic stresses. *BrMYB210*, *BrMYB137*, *BrMYB88*, *BrMYB154* and *BrMYB222* were significantly upregulated by both cold and osmotic stress treatments respectively (>10 fold-change), suggesting that they have roles in abiotic stress responses, much like their *Arabidopsis* orthologs. Similarly, the C11 subfamily members (consisting of 3R-type BrMYBs; *BrMYB3R5*, and *BrMYB3R9*) were also up-regulated by both stresses (Figure 7). Previous studies have revealed that plant MYB3R factors participate in the transcriptional control of cyclins, especially in late G_2_ and M phase, and OsMYB3R-2 regulates a cyclin involved in the CBF pathway to increase tolerance to low temperatures and drought [5,49]. Our results were consistent with these findings, and indicate that the 3R-MYB factors of Chinese cabbage are probably involved in stress response regulation, and that some homologous genes (e.g., *BrMYB3R5*-*BrMYB3R9*) may be functionally redundant in these processes. However, *BrMYB261* (an ortholog of *AtMYB28*) had no response to cold treatment, but was induced drastically by osmotic stress. Since *AtMYB28* was identified as a regulator of glucosinolate biosynthesis [50], a process involved in leaf water balance in broccoli [51], our findings strengthen the suggestion that *MYB28* homologs may be novel regulators in the plant water deficit response.

The plant hormones ABA and auxin control important cellular processes, including seed germination, leaf senescence, stomatal aperture and stress responses [52]. The qPCR results showed that 26 of the 43 *R2R3-MYB* genes were up-regulated under ABA treatment, most of which showed similar patterns to their *Arabidopsis* orthologs, suggesting that they function in the same processes [4]. For instance, *Arabidopsis* S1 subgroup members *MYB60* and *MYB96* act through the ABA signaling pathway to regulate stomatal movement and disease resistance [53]; likewise, the C36 (S1) members *BrMYB137* and *BrMYB210* had relatively high transcript levels in response to exogenous ABA, suggesting their roles in the ABA signaling cascade. Moreover, most of the 43 genes responded to auxin treatment. Among them, *BrMYB140*, *BrMYB172*, *BrMYB229*, *BrMYB208*, *BrMYB137* and *BrMYB210* were up-regulated by ABA but down-regulated by auxin, suggesting that these *R2R3-BrMYB* genes might act as regulators in ABA-auxin antagonistic regulation of senescence processes [54]. In addition, *AtMYB96* was shown to regulate lateral root meristem activation via ABA-auxin signaling crosstalk [55]. Notably, *BrMYB210* (an ortholog of *AtMYB96*) was down-regulated by auxin treatment in leaves; thus, we hypothesize that Chinese cabbage *MYB96*-homologous genes may also participate in ABA-auxin signaling crosstalk in the aerial parts of Chinese cabbage.

## Conclusions

In total, 256 (~55% of total BrMYBs) R2R3-MYB TFs were identified in the whole Chinese cabbage genome, most of which were localized on the 10 chromosomes and three subgenomes. Duplicated gene pairs among the *R2R3-BrMYB* genes were detected by syntenic analysis, which supports the genome triplication event in Chinese cabbage. Phylogenetic analysis of the R2R3-MYB family in Chinese cabbage and *Arabidopsis* revealed the conserved organization of this family, which further indicates that R2R3-MYB family members from various plants underwent gene duplication events with a common origin and were retained over a long period by each genome. Additionally, the increased number of R2R3-MYBs that seemingly evolved independently in Chinese cabbage and rice may contribute to plant viability under adverse conditions and functional specialization of R2R3-MYB genes. In addition, the tissue-specific expression profiles of the *R2R3-MYB* genes suggest that some of them have important roles in developmental and metabolic processes. Moreover, qPCR analysis indicated that several genes might function in stress responses and ABA-auxin hormone signal-mediated morphogenesis and cell senescence, which further highlights the functional diversity and indispensability of the *R2R3-MYB* genes in the normal growth and development of Chinese cabbage. This study gives an overview of the *R2R3-MYB* genes in Chinese cabbage and enabled us to provide some insights into plant stress response mechanisms and how transcription factors act in complex signal transduction, but how *R2R3-BrMYB* genes participate in these processes will require further investigation.

## Methods

### Identification of MYB transcription factors in different plants

The whole-genome proteins of Chinese cabbage were downloaded from BRAD (http://brassicadb.org/brad/) and those of other species used were obtained from PlantGDB (http://www.plantgdb.org). Then, the Pfam program was employed to search for candidate MYB genes in the extracted full-length protein sequences (http://pfam.sanger.ac.uk/). Only hits with e-values < 1.0 were considered to be members of the MYB family [56]. To confirm the obtained amino acid sequences, the putative MYB sequences were examined for the MYB domain using the hidden Markov model of the SMART tool (http://smart.embl-heidelberg.de/) and the ExPASy Proteomics Server (http://expasy.org/prosite/) [57]. Manual inspection was performed to ensure that the putative MYB genes contained conserved Trp (W) residues. The sequences of all MYB members in the genomes of other species assessed were downloaded from the plant TFDB database (http://planttfdb.cbi.edu.cn/). However, gene identifiers for 132 *Arabidopsis thaliana* R2R3- and R1R2R3-MYB genes were obtained from TAIR (http://www.arabidopsis.org/).

### Protein properties and conserved motif analysis

To investigate the protein properties of the putative BrMYB proteins, their molecular weights (MW) and isoelectric points (pI) were calculated using Pepstats (http://www.ebi.ac.uk/Tools/seqstats/emboss_pepstats/). The conserved motifs of the R2R3-MYB proteins were identified statistically with the MEME program (version 4.8.1) (http://meme.nbcr.net/meme/intro.html) [58]. The following parameter settings were used: maximum number of motifs, 50; minimum width of motif, 6; maximum width of motif, 250. All putative motifs with expected values < 1e-10 were discarded. Subsequently, the MAST program (version 4.8.1) (http://meme.nbcr.net/meme/cgi-bin/mast.cgi) was used to align the conserved motifs of the proteins.

### Multiple sequence alignment and phylogenetic analysis

Phylogenetic trees were produced individually using the full-length sequences of the R2R3-type MYB TFs. The DNA-binding domains (DBDs) of MYB proteins from *Arabidopsis*, Chinese cabbage and rice were subjected to multiple alignment analysis with ClustalW (http://www.ebi.ac.uk/Tools/msa/clustalw2/) and Weblogo analysis [59]. Phylogenetic analyses were conducted using MEGA5 (http://www.megasoftware.net/) with the Maximum-Likelihood (ML) and Neighbor-Joining (NJ) methods; the bootstrap value was set to 1000.

### Identification of orthologous and paralogous MYBs

The position of each BrMYB was marked on the chromosomes using a Perl script. The orthologous and paralogous MYB genes in Chinese cabbage and *Arabidopsis* were identified using OrthoMCl (http://orthomcl.org/orthomcl/). The relationships between the orthologous and paralogous genes among the three species were plotted using Circos (http://circos.ca/).

### Syntenic analysis and Ka/Ks calculation

The duplicated *R2R3-MYB* genes were identified using MCScanX (http://chibba.pgml.uga.edu/mcscan2/) as previously described [60]. The whole-genome protein sequences from Chinese cabbage were compared against each other using BLASTP, with a tabular output format and an e-value < 1e-20. The BLASTP results with simplified gene location files were used as an input for MCScanX to identify syntenic gene pairs and duplication types with default settings. We calculated the synonymous rate (Ks), non-synonymous rate (Ka) and evolutionary constraint (Ka/Ks) between the duplicated pairs of *R2R3-BrMYB*s (Table 1) based on their coding sequence alignments [61], and the divergence time was calculated according to the neutral substitution rate of 1.5 × 10^−8^ substitutions per site per year for chalcone synthase [62].

### RNA-seq data analysis

To analyze the Chinese cabbage *R2R3-MYB* expression patterns, we used Illumina RNA-seq data reported previously [39]. These data included three tissues (root, stem and leaf) of *B. rapa*. Gene expression levels were calculated as reads per kilobase of exon model per million mapped reads (RPKM) units (Additional file 8: Table S2). Heat maps were generated and hierarchical clustering was done using Cluster 3.0.

### Plant materials, growth conditions and stress treatments

Seedlings of Chinese cabbage cultivar YANZA03 were germinated in plastic Petri dishes in darkness at 22°C for 2 days, and then transferred to pots containing soil growth medium under artificial growth conditions of 22°C, approximately 120 μmol photons m^−2^ s^−1^, a photoperiod of 16/8 h, and 60% relative humidity. Half-strength Murashige and Skoog liquid solution (pH 5.8) was added once every 3 days. Five-leaf-stage plants were subjected to various treatments under a continuous time course (0, 12, 24, 48, and 96 h). For cold treatment, the pots were exposed to low temperature (4°C) conditions; for osmotic stress treatment, the pots were irrigated with 15% (w/v) polyethylene glycol (PEG) and kept standing in the irrigation solution for 30 minutes under normal growth conditions; hormone treatments were performed with ABA (100 μM) and auxin (50 mg/L NAA). The seedlings were harvested under a continuous time course (0, 12, 24, 48, and 96 h) with three biological replicates for RNA preparation.

### RNA isolation and quantitative real time-PCR (qPCR) analysis

Total RNA was isolated from treated leaves using Trizol (Invitrogen, San Diego, CA, USA) according to the manufacturer’s instructions. The total RNA was treated with DNase I (Invitrogen) and 1 μg treated RNA was reverse-transcribed using PrimeScript™ RT reagent Kit (Perfect Real Time) for qPCR (Takara, Dalian China). The *GAPDH* gene was used as an internal control [63]. The qPCR assays were performed with three biological and technical replicates. The SYBR® select Master Mix (Invitrogen) was used to detect gene expression according to the manufacturer’s recommendations on the One-step Real-Time PCR System (Applied Biosystems). qPCR was carried out according to a previous report [64]. Gene-specific primers that were used to detect transcripts are listed in Additional file 9: Table S4. The PCR conditions and relative gene expression calculation were as previously described [65].

### Availability of supporting data

The supporting sequence data are available in the Additional file 10: Table S5, and were obtained from *Brassica* database (http://brassicadb.org/brad/index.php). The supporting expression profile data are available in the Additional file 8: Table S2, and were obtained from a public data set (http://brassicadb.org/brad/genomeDominanceData.php).

## Additional files

Additional file 1: Table S1. Listing of MYB transcription factor genes in *Brassica rapa*. Additional file 2: Figure S1. Comparisons of DNA-binding domain of MYB-related and 3R-MYB transcription factor proteins in Arabidopsis, Chinese cabbage and rice. Additional file 3: Figure S2. Distribution of MYB genes on 10 chromosomes and 3 subgenomes. Additional file 4: Figure S3. Phylogenetic relationships and subgroup designations in MYB proteins from Chinese cabbage and Arabidopsis. Additional file 5: Figure S4. The NJ tree of R2R3-MYBs from Arabidopsis and Chinese cabbage. Additional file 6: Table S3. The statistic analysis of each group of ML tree. Additional file 7: Figure S5. ML phylogenetic tree of R2R3-MYBs from Arabidopsis, Chinese cabbage and rice. Additional file 8: Table S2. The genomic distribution and RPKM values of R2R3 MYB gene family in Chinese cabbage. Additional file 9: Table S4. Primers for quantitative PCR of R2R3-BrMYBs. Additional file 10: Table S5. Listing of BrMYB gene sequences.
