# Supplementary material for: Genome-wide analysis of the R2R3-MYB transcription factor genes in Chinese cabbage (Brassica rapa ssp. pekinensis) reveals their stress and hormone responsive patterns
Source: BMC Genomics. 2015 Jan 23;16(1):17. doi: 10.1186/s12864-015-1216-y (PMC4334723; doi:10.1186/s12864-015-1216-y)
Supplement: Additional file 2: Figure S1. — Comparisons of DNA-binding domain of MYB-related and 3R-MYB transcription factor proteins in Arabidopsis, Chinese cabbage and rice. [file 12864_2015_1216_MOESM2_ESM.pdf]

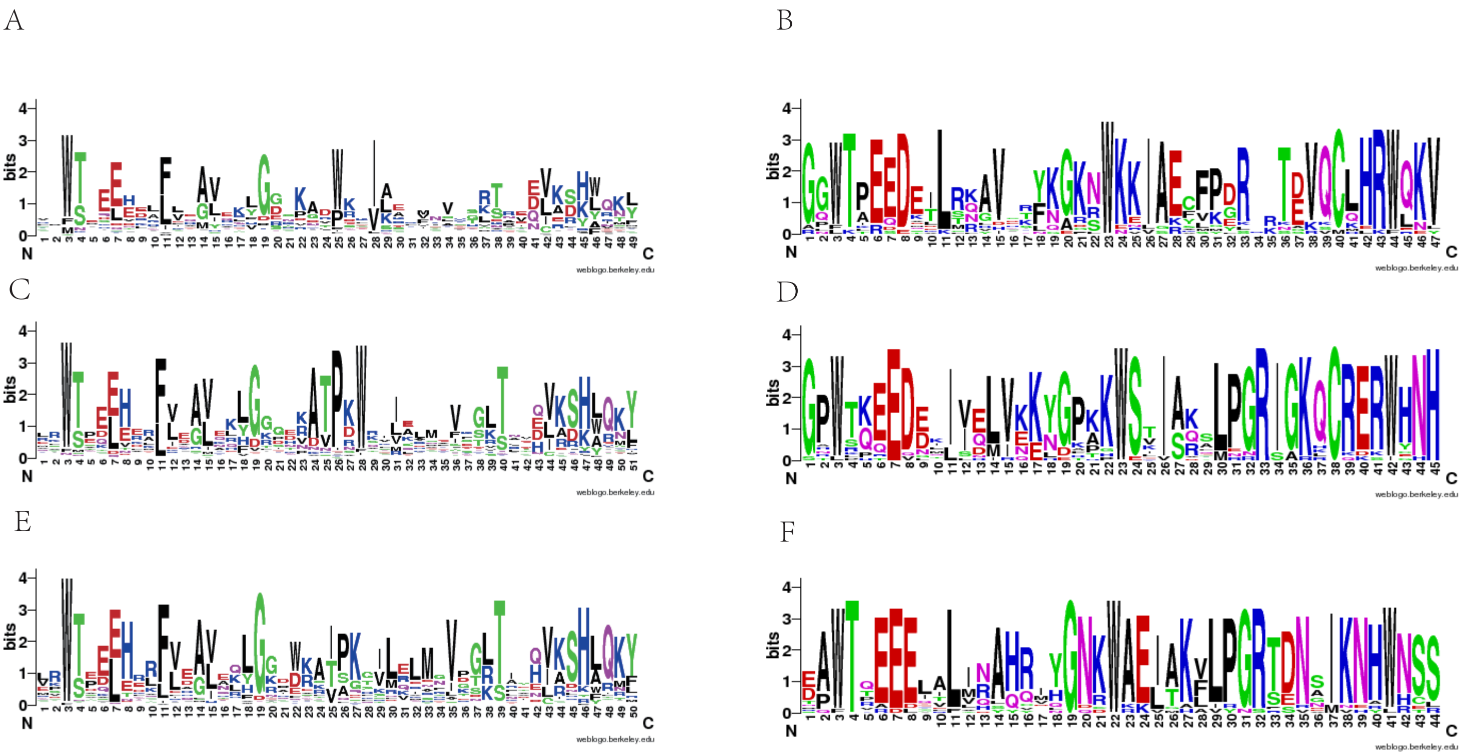
A
B
C
D
E
F
bits
N
C
weblogo.berkeley.edu
