# Supplementary figures and images for: Genome-wide analysis of the R2R3-MYB transcription factor genes in Chinese cabbage (Brassica rapa ssp. pekinensis) reveals their stress and hormone responsive patterns
Source: BMC Genomics. 2015 Jan 23;16(1):17. doi: 10.1186/s12864-015-1216-y (PMC4334723; doi:10.1186/s12864-015-1216-y)

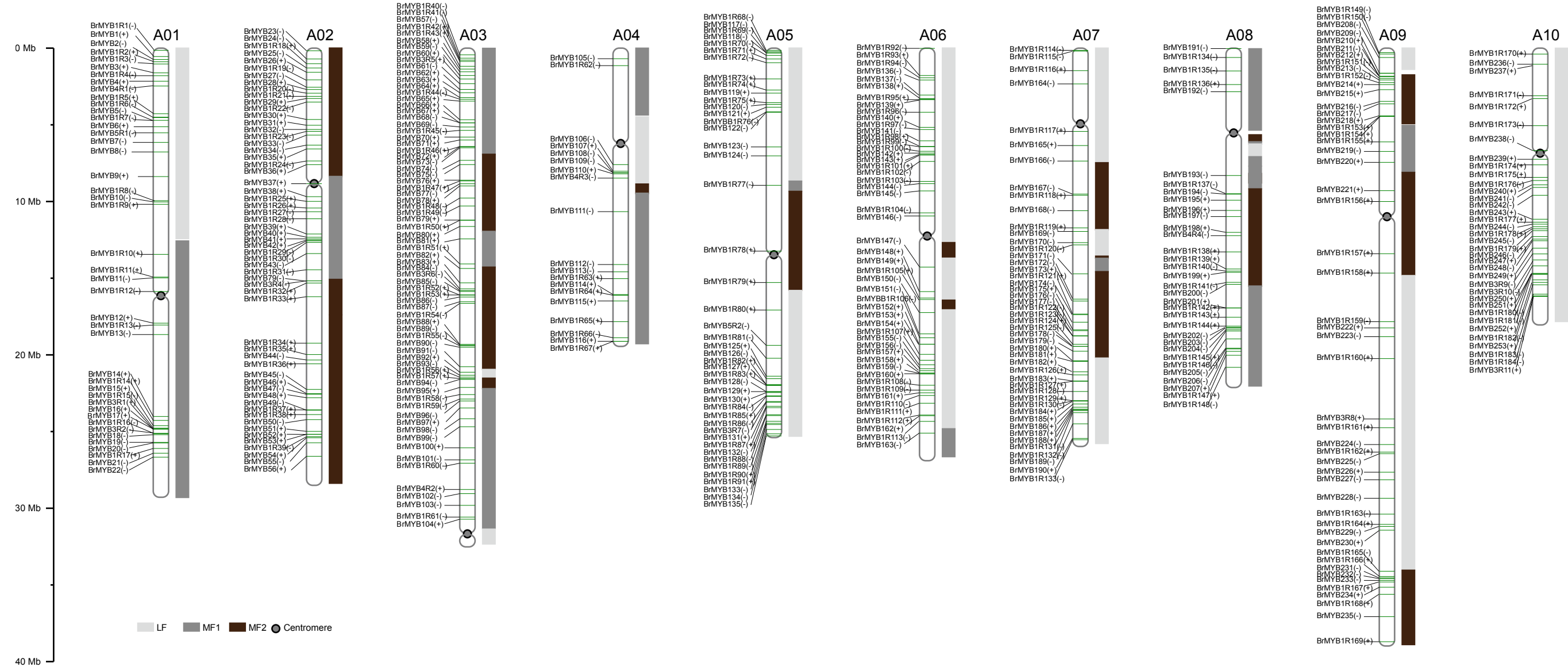

Supplement: Additional file 3: Figure S2. — Distribution of MYB genes on 10 chromosomes and 3 subgenomes. [file 12864_2015_1216_MOESM3_ESM.pdf]

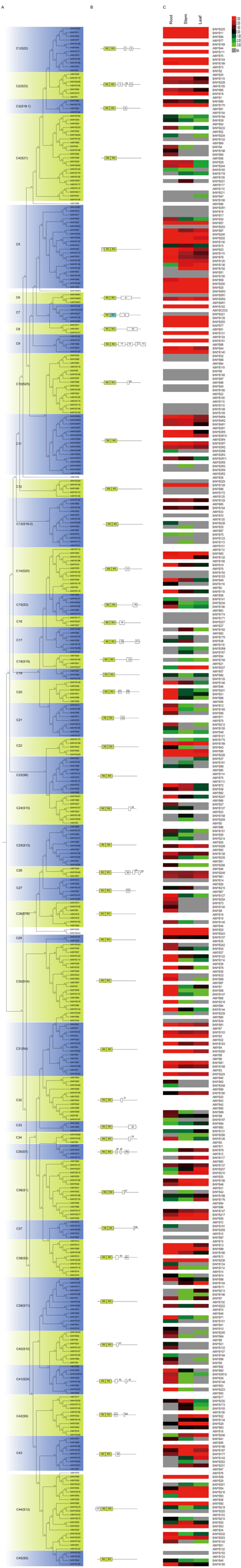

Supplement: Additional file 4: Figure S3. — Phylogenetic relationships and subgroup designations in MYB proteins from Chinese cabbage and Arabidopsis. [file 12864_2015_1216_MOESM4_ESM.pdf]

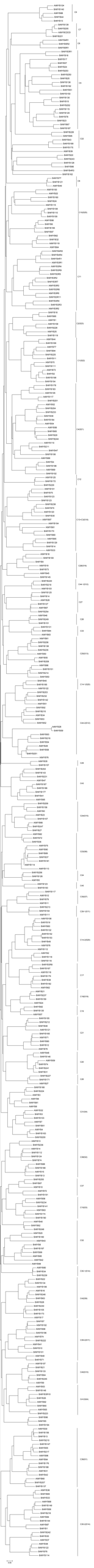

Supplement: Additional file 5: Figure S4. — The NJ tree of R2R3-MYBs from Arabidopsis and Chinese cabbage. [file 12864_2015_1216_MOESM5_ESM.pdf]

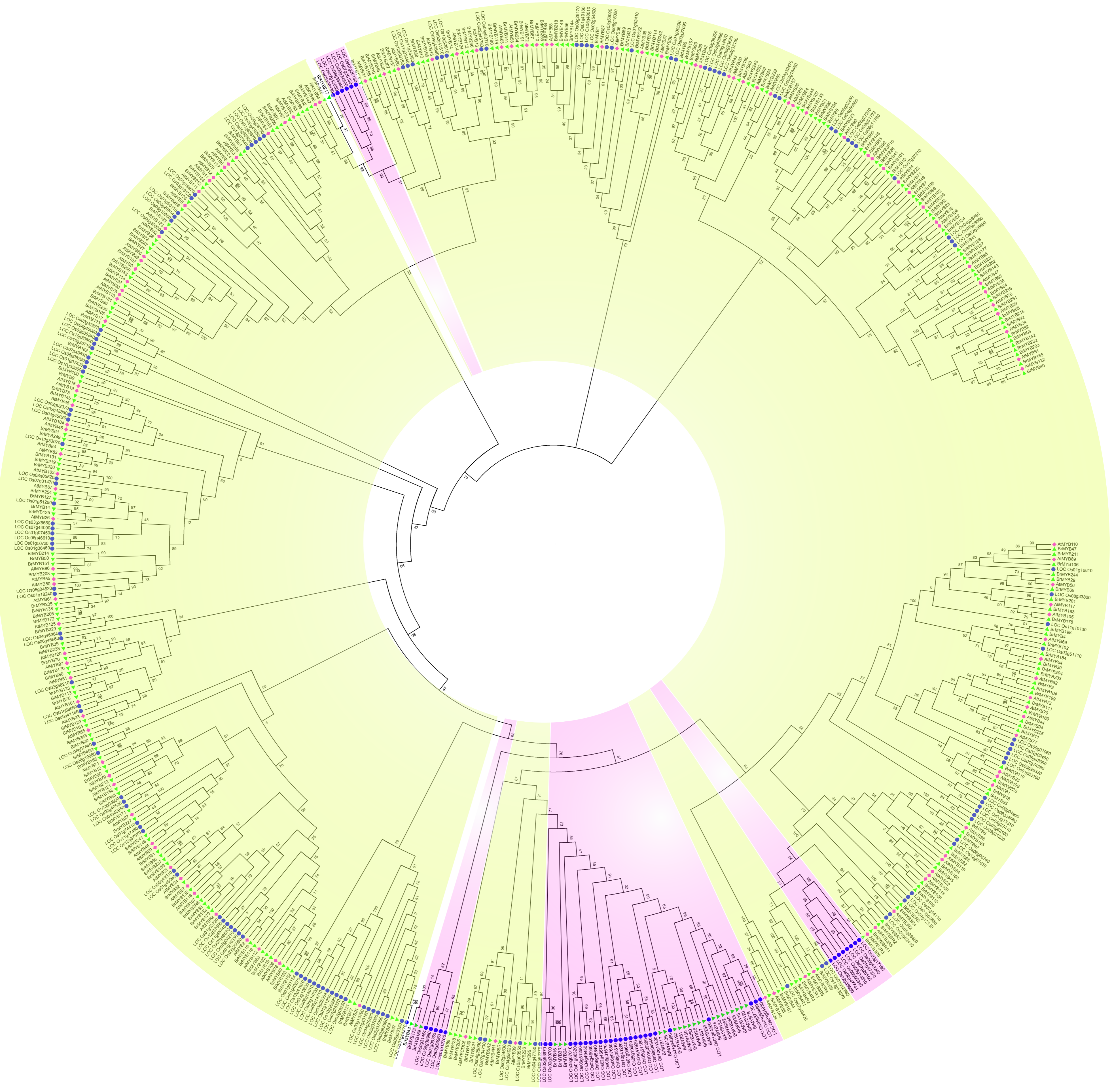

Supplement: Additional file 7: Figure S5. — ML phylogenetic tree of R2R3-MYBs from Arabidopsis, Chinese cabbage and rice. [file 12864_2015_1216_MOESM7_ESM.pdf]
